# Fourier Series Analysis for Novel Spatiotemporal Pulse Waves: Normal, Taut, and Slippery Pulse Images

**DOI:** 10.1155/2019/5734018

**Published:** 2019-11-27

**Authors:** Bo Peng, Ching-Hsing Luo, Nilotpal Sinha, Cheng-Chi Tai, Xiaohua Xie, Haiqing Xie

**Affiliations:** ^1^Department of Electrical Engineering, National Cheng Kung University, Tainan 70101, Taiwan; ^2^School of Data and Computer Science, Sun Yat-Sen University (East Campus), Guangzhou 510006, China; ^3^School of Medical Engineering, Foshan University, Foshan 528000, China

## Abstract

In this article, a three-dimensional pulse image (3DPI) instead of a one-dimensional temporal pulse wave is studied to elucidate its spatiotemporal characteristics. To check the spatial and temporal properties of 3DPI, adopted is Fourier series, in which a ratio (*r*) is defined as one amplitude divided by the sum of the first three amplitudes of harmonics. A ratio sequence is constituted from 70 to 90 ratios in a heartbeat with 70–90 3DPIs by sampling. Twenty-four subjects (14 males and 10 females with age of 22.2 ± 3.7 years, 20.4 ± 1.4 BMI, and 112.1 ± 4.7 mmHg systolic blood pressure) are involved in this research. There are significant statistical differences in the groups of the normal, taut, and slippery 3DPIs by the first harmonic ratio average ($\bar{r_{1}}$) and ratio difference (Δ*r*_1_) produced from the ratio sequence. The proposed method of this study gives us a novel viewpoint to clarify the spatiotemporal characteristics of pulse images, which can translate and quantize the pulse feeling in Chinese medicine texts.

## 1. Introduction

Chinese medicine has been developed for over two thousand years. Pulse diagnosis in Chinese medicine provides a noninvasive method based on the radial pulse feeling of the fingers with temporal and spatial characteristics. In order to measure and analyze these pulses, several pulse diagnostic instruments (PDIs) were developed based on array sensors for detecting not only the temporal characteristics but the spatial characteristics of the pulse [1–3]. Luo and colleagues [4–6] used the PDI with tactile array sensors to get the radial artery pressure signal transformed into a visualized three-dimensional waveform, called a three-dimensional pulse image (3DPI). Three types of pulse feeling have been well transformed into 3DPI: normal pulse, taut pulse, and slippery pulse [7].

The signal analysis of 3DPI has been gradually paid attention in recent years. Cui et al. [8] proposed a method for distinguishing taut pulse from normal and slippery pulse based on array pulse volume (APV), defined as the average volume of signals in a pulse cycle. Su et al. [9] suggested to conveniently distinguish normal and taut pulses by contour analysis of the spatial structure, and Luo et al. [10] proposed the L-cube polynomial to describe the 3DPI patterns of these two types.

There are a lot of analytic methods in single pulse wave analysis (PWA) [11–24], of which fast Fourier transform (FFT), Hilbert–Huang transform (HHT), wavelet transform (WT), etc. are efficient ways of viewing waveforms in frequency or time domain. However, it is shown that Fourier series can well present the component of pulse waves given the periodicity of the cardiovascular system [25–30]. Since the pulse signal in a short period of time can be treated as a periodic signal due to its small nonlinearity [27, 31], Fourier series is considered a unique and powerful pulse analysis method. Given that the spatial properties of the pulse need to be further analyzed, this study intends to determine the features of three pulse types using Fourier series expanding from one-dimensional to three-dimensional pulse analysis.

## 2. Materials and Methods

### 2.1. Experimental Data

The pulse data in this research were collected from healthy subjects using the PDI (IRB no: A-BR-100-166). The subjects included 14 males and 10 females from college students (Table 1). The target three types of pulse were first verified by Chinese medicine practitioners' palpation based on the Chinese medicine theory [32], and finally confirmed by the peripheral augmentation index (pAI), which was defined as the ratio of percussion and tidal wave as well as regularly used in Chinese medicine pulse pattern research [9, 21, 33–35]. Normal pulse data were obtained from these subjects in normal state, taut pulse images were induced by cold pressor test (CPT) [9, 36, 37], while slippery pulse images were measured from woman subjects during the menstrual cycle [7]. The pulse wave signals at the left wrist on a participant were recorded by the tactile array sensor on position “Guan” of the PDI [1]. The PDI thus continuously recorded 12-channel pulse wave signals during the CPT for each participant. There are three robot fingertips in the PDI with 12-point (3 × 4) sensors on their tips, and each 12-point tactile sensor array has the size of 1 cm × 0.75 cm with 100 Hz sampling rate [1, 5, 7, 38].

### 2.2. Experimental Procedure

**Figure 1:**
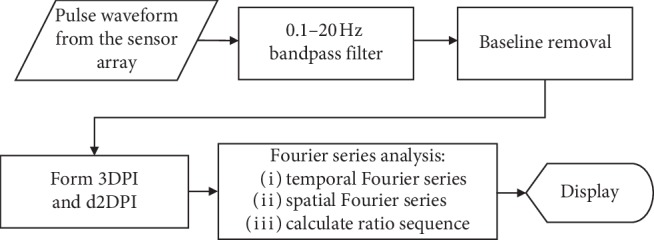
Flowchart for pulse image processing.

A flowchart (Figure 1) is given to show the way to conduct pulse image analysis. First, we took the readings from the sensor arrays in the form of a column matrix with a sampling frequency of 100 Hz. The 0.1–20 Hz bandpass Butterworth filter was used to reduce the noise, and baseline drift was greatly decreased by wavelet transform [38, 39]. 3DPI is formed by using a cubic interpolation algorithm to expand graphic data points [40], and a two-dimensional pulse image (2DPI) is formed from 3DPI by taking the middle pulse data along the center of the radial artery. Fourier series was adopted to analyze the radial pulse images.

**Figure 2:**
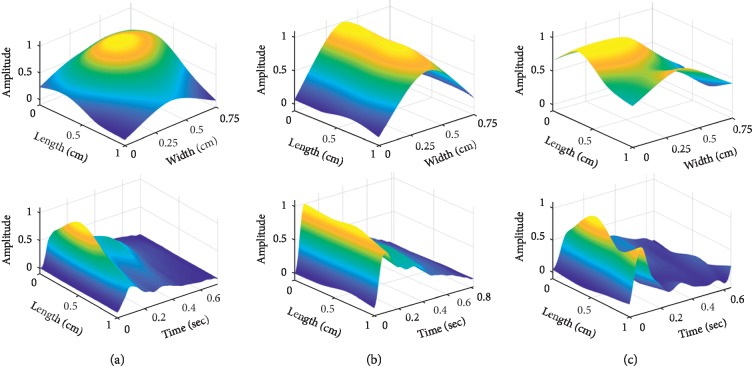
Multidimensional pulse images: (a) normal pulse, (b) taut pulse, (c) and slippery pulse: upper panel, 3DPI; lower panel, d2DPI.

Figure 2 shows a multidimensional pulse image: 3DPI (upper panel) at the peak pulse and d2DPI (dynamic 2DPI, lower panel) of one beat for (a) normal, (b) taut, and (c) slippery pulses. The d2DPI clearly demonstrates the temporal response with one spatial information of array pulse signals, while 3DPI just presents two spatial data without temporal responses in the figure.

**Figure 3:**
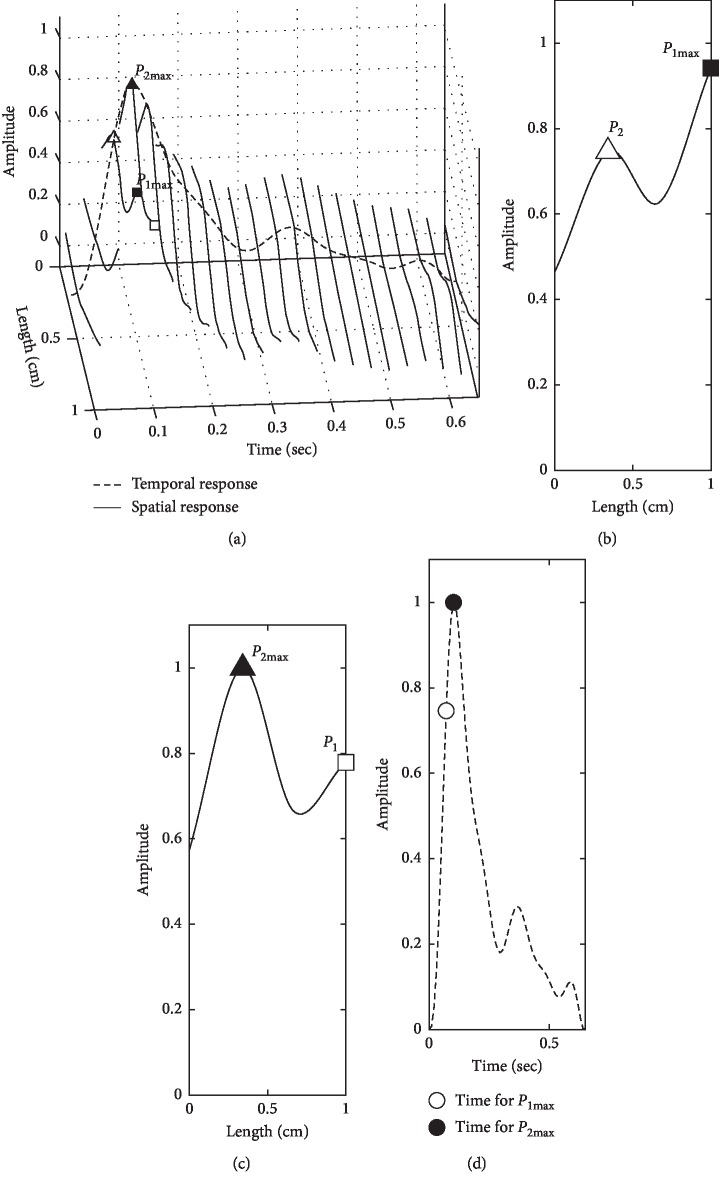
Temporal and spatial waves of a slippery d2DPI. (a) Temporal and spatial waves shown in a single pulse wave. (b) The first spatial peak maximum (*P*_1max_, black rectangle). (c) The second spatial peak maximum (*P*_2max_, black triangle). (d) Time for *P*_1max_ (white circle) and *P*_2max_ (black circle) in a temporal wave.

In Figure 3(a), d2PDI of a slippery wave is further clearly demonstrated by temporal and spatial responses separately, in which the temporal pulse in one beat is same as the regular single pulse wave, and the wave shape of the spatial pulse is presented along the center of the radial artery. Two spatial peaks move up and down alternately in a slippery 3DPI shown in Figure 3 marked with a triangle and a rectangle.

The first peak moves to its maximum (*P*_1max_) at length = 1 in Figure 3(b) (black rectangle) and time = 0.07 in Figure 3(d) (white circle), while the second peak reaches its maximum (*P*_2max_) at length = 0.34 in Figure 3(c) (black triangle) and time = 0.10 in Figure 3(d) (black circle). It is especially noted that only one peak exists in the temporal wave while two peaks appear in the spatial wave for a slippery 3DPI or d2DPI.

### 2.3. Primary Algorithm

A general Fourier series analysis is applied to calculate the temporal or spatial harmonic components of normal, taut, and slippery d2DPIs shown below:

(1) $$\begin{array}{c} f\left(x\right)=\overset{\infty }{\underset{n=\;-\infty }{\sum }}C_{n}e^{j\left(2\pi nx/X\right)}\;, \end{array}$$

where

(2) $$\begin{array}{c} C_{n}=\;\frac{1}{X}\int_{0}^{X}f\left(x\right)e^{-j\left(2\pi nx/X\right)}\text{d}x. \end{array}$$

*C*_*n*_ is the amplitude of the temporal or spatial harmonics for *x* and *X* = time or length separately.

Fourier series with seven or eight harmonics is empirically adequate for 99.5% of the variance for one-dimensional temporal wave [41, 42], while the qualified harmonic number of spatial Fourier series is educed to three, on account of the simplicity of the spatial wave shape in this study.

## 3. Results and Discussion

### 3.1. Temporal Fourier Series

**Figure 4:**
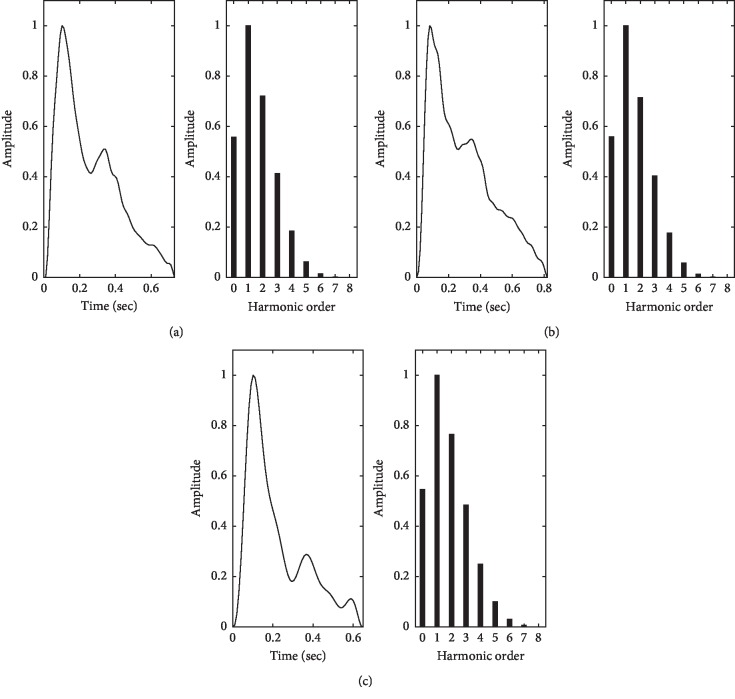
(a) Normal, (b) taut, and (c) slippery pulse's temporal wave (left) and their Fourier series (right).

Figure 4 shows the amplitudes of the temporal Fourier series for normal, taut, and slippery pulse images separately. Even there is a little variation in these three temporal pulse waves, but their harmonic amplitudes make no remarkable changes.

### 3.2. Spatial Fourier Series

**Figure 5:**
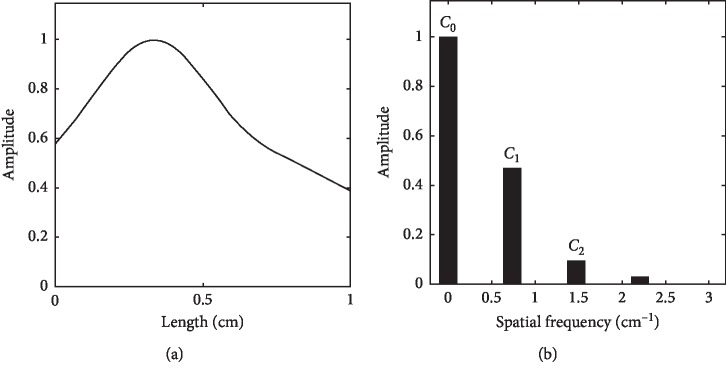
Normal pulse spatial wave (a) and its Fourier series (b).

**Figure 6:**
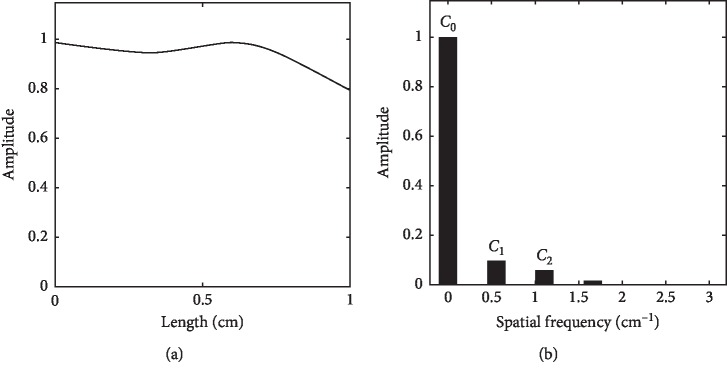
Taut pulse spatial wave (a) and its Fourier series (b).

Figures 5–6 presents the amplitudes of spatial Fourier series for normal and taut pulse images. By neglecting the base amplitude at 0 Hz (*C*_0_), the first (*C*_1_) and second (*C*_2_) harmonics show a remarkable difference in amplitudes between normal and taut pulse images. *C*_1_ is much bigger than *C*_2_ at normal pulse image, while *C*_1_ is almost equal to *C*_2_ at taut pulse image. At taut pulse image, most of the amplitude is accumulated at the base because the taut pulse image looks like a rectangular pulse with little harmonics in the spatial length along the radial artery.

**Figure 7:**
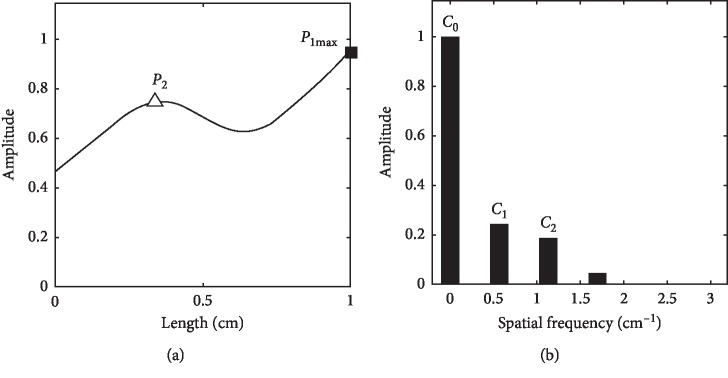
Slippery pulse spatial wave with the first peak (a) and its Fourier series (b).

**Figure 8:**
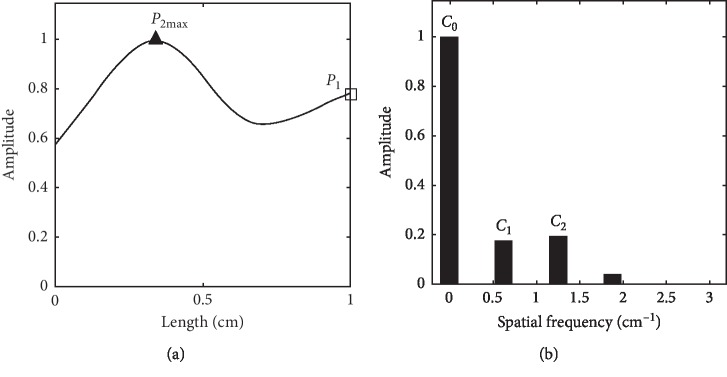
Slippery pulse spatial wave with the second peak (a) and its Fourier series (b).

Figures 7–8 show the spatial Fourier series at the first and second peak maximums (*P*_1max_ and *P*_2max_) of slippery pulse images separately. For the spatial wave at *P*_1max_, its *C*_1_ is greater than *C*_2_ in Figure 7, while for the spatial wave at *P*_2max_, its *C*_1_ becomes smaller than *C*_2_ in Figure 8.

### 3.3. Spatial Harmonic Ratio Sequence

To make the recognition among these three pulse images, harmonic ratios (*r*_0_, *r*_1_, and *r*_2_) are defined as follows:

(3) $$\begin{array}{c} {\text{r}}_{0}=\frac{C_{0}}{C_{0}+C_{1}+C_{2}},\\ {\text{r}}_{1}=\frac{C_{1}}{C_{0}+C_{1}+C_{2}},\\ {\text{r}}_{2}=\frac{C_{2}}{C_{0}+C_{1}+C_{2}}, \end{array}$$

where *C*_0_, *C*_1_, and *C*_2_ are the amplitudes of the base, first, and second harmonics in the spatial Fourier series.

**Figure 9:**
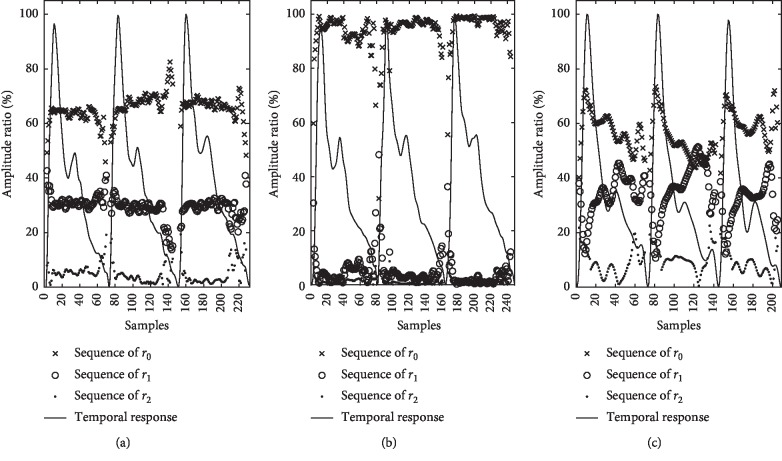
Spatial harmonic ratio sequences of the Fourier series amplitudes: (a) normal pulse, (b) taut pulse, and (c) slippery pulse.

For a cardiac cycle, we can get a spatial harmonic ratio sequence from d2DPI.  Figure 9 demonstrates three spatial harmonic ratio sequences of consecutive three beats for normal, taut, and slippery pulses. To avoid noise interference at small amplitudes, ratio sequences are not calculated for the pulse with its peak less than 5% of peak maximum [27]. The harmonic ratio sequence of normal pulses in Figure 9(a) is well distributed without any intersection. For taut pulses in Figure 9(b), the base ratio sequence is always far beyond the first and second harmonic ratio sequences which are almost merged together. Regarding slippery pulses in Figure 9(c), its first and second harmonic ratio sequences intersect each other at the upstroke phase due to two peaks oscillating alternatively. Occasionally, the first harmonic ratio sequence becomes high enough to interact with the base harmonic ratio sequence. Apparently, the behavior of three harmonic ratio sequence showing remarkable differences creates the capability of the recognition among normal, taut, and slippery pulse images (Table 2).

### 3.4. Statistics

**Figure 10:**
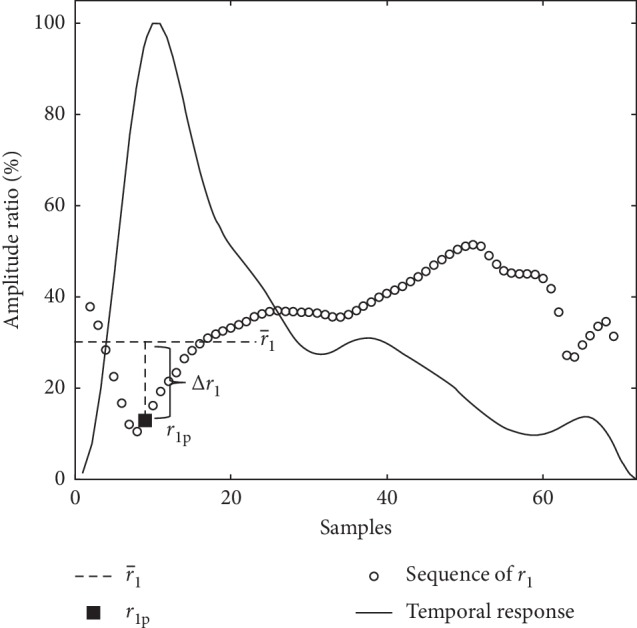
The relationship between the $\bar{r_{1}}$, *r*_1p_, and Δ*r*_1_ in a slippery pulse.

In this study, the first harmonic ratio average $\bar{r_{1}}$ is adopted to represent spatial features of the pulse image, which is the average of *r*_1_ sequence in one heartbeat. The ratio difference Δ*r*_1_ is provided by the difference between $\bar{r_{1}}$ and the *r*_1_ at the peak of the pulse (*r*_1p_), which is more obvious in slippery cases than others (Figure 10). For comparison, pAI [33], array pulse volume (APV) [8], 2DL from contour analysis [9], and *r*_Lc_ from L-cube polynomial [10] are listed in Table 3.

All the indexes are calculated for pulse datasets with normalized amplitude from 0 to 1 and analyzed using one-way analysis of variance (ANOVA). A *P* value less than 0.05 is considered statistically significant (Table 4).

According to statistical analysis, APV is unable to entirely differentiate taut and slippery pulses (2.30 ± 0.33 vs. 2.18 ± 0.13; *P*=0.2809 > 0.05) by losing the spatial characteristics and trend of 3DPI. 2DL and *r*_Lc_ which include spatial characteristics of 3DPI, respectively, have significant statistical differences in normal and taut pulses, but they are not designed for describing the pulse's spatial waves containing more than one peak like a slippery pulse.

Apparently, normal, taut, and slippery pulse images can be differentiated by $\bar{r_{1}}$ with spatial characteristics of 3DPI. On the side, Δ*r*_1_ which reflects the property of spatiotemporal changes is also a recommendable index that well differentiates slippery pulse images from normal and taut pulse images.

As an arterial stiffness index of a single temporal pulse wave [35], our results in Table 3 agree with the previous classification research that the pAI value of a normal pulse is higher than that of a slippery pulse while lower than that of a taut pulse [33]. It is worth noting that a taut pulse and a tight pulse in Chinese medicine are in similar shape and related to vessel tensing [32]. It is suggested that CPT on a healthy subject's hand and wrist [9, 36, 37] can briefly increase arterial stiffness to induce a taut pulse, not a tight pulse, which fairly presents illness.

## 4. Conclusions

Fourier series is provided as a fast, robust, and real-time able method to analyze 3DPIs. It is difficult to differentiate these three types of pulses (normal, taut, and slippery) for the temporal Fourier series only because the definition of pulse feeling in Chinese medicine takes the spatial waveshape factor into account [1, 5, 7]. In comparison with temporal Fourier analysis in Table 2, the spatial Fourier analysis can distinguish among normal, taut, and slippery pulse images. For the Fourier series analysis of three spatiotemporal 3DPIs (normal, taut, and slippery), the proposed harmonic ratio sequence clearly shows apparently different behavior among three pulse images, and there are statistical differences between them by $\bar{r_{1}}$ and Δ*r*_1_ indexes.

In this study, taut pulses were induced by CPT, not collected from hypertensive patients. Normal and slippery pulse images were obtained from young people. In the near future, lots of 3DPI should be collected from patients with different ages and several kinds of hypertension to testify the statistical recognition rate of the proposed ratio sequence. Furthermore, various methods such as WT and HHT can be introduced to study the spatiotemporal characteristics of all types of 3DPI, not limited only with normal, taut, and slippery pulse images.

## Figures and Tables

**Table 1:** Basic physiological data of the subjects.

Characteristic (unit)	Number or mean ± SD
Number	24 (male = 14, female = 10)
Age (years)	22.2 ± 3.7
BMI (kg/m^2^)	20.4 ± 1.4
Systolic/diastolic blood pressure (mmHg)	112.1/74.0 ± 4.7/3.4
Heart rate (beats/min)	76.0 ± 5.4

**Table 2:** Fourier series analysis of normal, taut, and slippery pulses.

Pulse type	Fourier series analysis of spatiotemporal pulse waves		
	Temporal analysis	Spatial analysis	Spatial harmonic ratio sequence
Normal pulse	Undistinguishable	Base and first harmonic share importance	Three sequences are well distributed
Taut pulse		Behaves as a rectangular pulse with dominant base and little harmonics	Base is high, and others merged together
Slippery pulse		The first harmonic can be higher or lower than the second one	Three sequences intersect each other

**Table 3:** Spatiotemporal pulse statistics.

Methods and indexes	Normalized value		
	Normal pulse	Taut pulse	Slippery pulse
pAI^*∗*^ [33]	0.65 ± 0.05	0.75 ± 0.07	0.50 ± 0.03
Array pulse volume [8]	1.85 ± 0.22	2.30 ± 0.33	2.18 ± 0.13
2DL from contour analysis [9]	0.45 ± 0.06	0.75 ± 0.11	—
*r*_Lc_ from L-cube polynomial [10]	1.21 ± 0.15	2.14 ± 0.44	—
[$\bar{r_{1}}$, Δ*r*_1_] from the proposed method	[0.39 ± 0.04, 0.00 ± 0.02]	[0.25 ± 0.05, 0.03 ± 0.04]	[0.32 ± 0.03, 0.16 ± 0.03]

^*∗*^pAI is calculated from a single temporal pulse wave, not a spatial pulse wave.

**Table 4:** *P* value from ANOVA test.

Indexes	*P* value		
	Normal-taut	Taut-slippery	Slippery-normal
pAI	0.0016	0.0000	0.0000
APV	0.0014	0.2809	0.0006
2DL	0.0000	—	—
*r*_Lc_	0.0000	—	—
$\bar{r_{1}}$	0.0000	0.0009	0.0002
Δ*r*_1_	0.0274	0.0000	0.0000

## Data Availability

The data used to support the findings of the current study are available from the corresponding author on reasonable request.
